# An 8-Hydroxy-Quinoline Derivative Protects Against Lipopolysaccharide-Induced Lethality in Endotoxemia by Inhibiting HMGB1-Mediated Caspase-11 Signaling

**DOI:** 10.3389/fphar.2021.673818

**Published:** 2021-05-21

**Authors:** Xiangyu Wang, Jian Shi, Zhaozheng Li, Ling Li, Rui Zhang, Yang Bai, Junmei Li, Fang Liang, Yiting Tang

**Affiliations:** ^1^ Department of Physiology, School of Basic Medical Science, Central South University, Changsha, China; ^2^ Department of Hematology and Critical Care Medicine, The Third Xiangya Hospital, Central South University, Changsha, China; ^3^ Department of Spine Surgury, The Third Xiangya Hospital, Central South University, Changsha, China

**Keywords:** caspase-11, pyroptosis, inflammasome, phenotypic screening, sepsis, HMGB1

## Abstract

Sepsis, an inflammatory syndrome secondary to infection, is the leading cause of in-hospital lethality. It is evidenced that LPS, the major pathological component of the Gram-negative bacteria membrane, predominantly contributes to the pathogenesis of sepsis. Cytoplasmic lipopolysaccharide (LPS) can be sensed by the noncanonical inflammasome and triggers the oligomerization of caspase-11, resulting in pyroptosis and lethal immune responses in sepsis. A previous study has shown that hepatocyte-released high mobility group box 1 (HMGB1) mediates caspase-11–dependent lethality in sepsis by delivering extracellular LPS into the cytosol. Here, we established a phenotypic screening system using recombinant HMGB1 plus LPS in mouse peritoneal macrophages, identifying a novel 8-hydroxyquinoline derivative named 7-[phenyl (pyridin-2-ylamino) methyl] quinolin-8-ol (8-ol, NSC84094) that can specifically inhibit HMGB1-mediated caspase-11 signaling. 8-ol targets directly to HMGB1 and changes the secondary conformation, consequently disrupting the interaction between LPS and HMGB1 and inhibiting the HMGB1-mediated delivery of LPS into the cytosol. Intervention of 8-ol significantly reduced the release of IL-1α and IL-1β and protected against caspase-11–mediated organ injury and lethality in endotoxemic mice. Thus, this study clearly suggests that the HMGB1–caspase-11 pathway is a potential drug target in lethal immune disorders and might open a new avenue in the treatment of sepsis.

## Introduction

Sepsis, caused by dysregulated host responses to infection, is a syndrome accompanied by life-threatening organ dysfunction (Singer et al., 2016). Thanks to the advance of life-supporting techniques, the mortality of sepsis has been decreasing in recent decades. Nevertheless, sepsis is still the leading cause of death in in-patient departments of hospitals (Rudd et al., 2020). Epidemiologically, it is reported that the prevalence of sepsis worldwide reached up to 48.9 million in 2017, among which 11.0 million patients died. Lipopolysaccharide (LPS), the major component of the Gram-negative bacteria outer membrane, results in dysregulated immune responses in sepsis (Angus and van der Poll, 2013). Removal of LPS is beneficial for patients with sepsis (Ronco et al., 2010). Thus, targeting LPS-triggered immune responses may be a promising strategy for treating sepsis.

Caspase-11 is a receptor of cytosolic LPS and triggers noncanonical inflammasome activation independent of TLR4, the extracellular receptor of LPS (Kayagaki et al., 2011; Kayagaki et al., 2013; Shi et al., 2014; Kayagaki et al., 2015). Intracellular LPS directly binds to caspase-11 (human orthologs caspase-4 or -5), which induces the oligomerization and activation (Shi et al., 2014). Activated caspase-11 cleaves GSDMD and releases pore-forming N-terminal GSDMD, triggering a programmed lytic cell death (pyroptosis) and interleukin 1 release (Shi et al., 2015; Liu et al., 2016; Evavold et al., 2018). In addition, activation of GSDMD mediates phosphatidylserine exposure, boosts the coagulation cascade, and promotes the process of disseminated intravascular coagulation (DIC), a complication mediating organ dysfunction or even death in sepsis (Levi & Ten Cate, 1999; Dhainaut et al., 2004; Zeerleder et al., 2005; Yang et al., 2019; Iba & Levy, 2020). A series of studies have shown that deficiency of caspase-11 and GSDMD protects against lethal endotoxemia (Kayagaki et al., 2011; Kayagaki et al., 2013; Shi et al., 2014; Kayagaki et al., 2015; Shi et al., 2015) and prevents DIC in sepsis (Yang et al., 2019). Our previous study found that hepatocyte-released HMGB1 delivers extracellular LPS into the cytosol of macrophages and mediates caspase-11–dependent pyroptosis in sepsis (Deng et al., 2018). Inhibition of HMGB1–LPS binding and neutralizing extracellular HMGB1 prevents caspase-11–dependent pyroptosis and death in endotoxemia (Deng et al., 2018).

In light of our previous study, we established a phenotypic screening strategy using recombinant HMGB1 plus LPS in mouse peritoneal macrophages. By screening large amounts of chemical compounds, we identified a novel 8-hydroxyquinoline derivative that specifically inhibited HMGB1-mediated caspase-11 signaling. Mechanistically, it suppresses the HMGB1–LPS interaction by changing the secondary conformation of HMGB1 and consequently inhibits the delivery of LPS. Treatment with the 8-hydroxyquinoline derivative protects mice against caspase-11–dependent lethality in endotoxemia and sepsis. Collectively, this study provides a novel drug-screening system and a new avenue for treating sepsis, which may facilitate the therapeutic strategy of sepsis.

## Materials and Methods

### Reagents and Antibodies

Recombinant HMGB1 protein was kindly provided by Dr. Kevin J. Tracey.

Ultrapure LPS (*E. coli* 111:B4, tlrl-3pelps), nigericin (tlrl-nig), ATP (tlrl-atp), MSU crystals (tlrl-msu), nano-SiO_2_ (tlrl-sio), FLA-ST (flagellin from *Salmonella typhimurium*) (Cat No. tlrl-stfla), and poly (dA:dT) naked (Cat No. tlrl-patn) were purchased from InvivoGen. Lipofectamine 3000 Transfection Reagent (Cat No. L3000015) was obtained from Thermo Fisher (Waltham, MA, United States). Digitonin (D141) was obtained from Sigma.

Antibodies were as follows: caspase-11 (Novus Biologicals, NB120), GSDMD (Abcam, ab209845), IL-1α (Abcam, ab7632), caspase-1 (Abcam, ab179515), IL-1β (R&D System, AF-401-NA), human GSDMD (Abcam, ab210070), cleaved N-terminal GSDMD (ab215203), IL-1α (Abcam, ab206410), IL-1β (Abcam, ab216995), caspase-4 (MBL International, M029-3), LAMP1 (eBioscience, 14-1071-85), Na^+^/K^+^ ATPase (Novus Biologicals, NB300-146), Rab7 (Cell Signaling Technology, Inc., #9367S), *E. coli* LPS antibodies (Abcam, ab35654), and *β*-actin (Cell Signaling Technology, Inc., #3700s).

### Animals

NLRP3^−/−^ mice were kindly provided by Professor Rongbin Zhou. *Casp11* ^−/−^, TLR4^−/−^ mice were purchased from Jackson Laboratory. In the current study, we used WT littermates as the controls for the transgenic mice. The animals were housed in a specific pathogen-free environment at the Department of Laboratory Animals of Central South University. All experimental animal protocols were approved by the Institutional Animal Care and Use Committees of Central South University.

### Endotoxemia Model

Mice aged 8–10 weeks were intraperitoneally pretreated with 4 mg/kg of the inhibitor 8-ol 30 min before being intraperitoneally injected with 20 mg/kg of LPS (*E. coli* O111:B4, Sigma). The mice were monitored 4 times daily for a total of 5 days or sacrificed at 16–24 h after LPS injection to collect serum or plasma and tissues.

### CLP Bacterial Sepsis Model

A cecal ligation and puncture (CLP)–induced polymicrobial sepsis model was utilized as previously described (Deng et al., 2018). Briefly, the mice were anesthetized by intraperitoneal injection with 2.5% tribromoethanol (Sigma). The skin was disinfected with a 2% iodine tincture, and a midline laparotomy was performed. The cecum was 75% ligated and punctured once with an 18-gauge needle. The mice received a subcutaneous injection of warm sterile saline (1 ml) immediately after surgery for fluid resuscitation. 8-ol (4 mg/kg) was administered 1h after CLP. The mice were monitored 4 times daily for 7 consecutive days or euthanized at 18–20 h after CLP to collect serum or lung tissue.

### Macrophage Cell Cultures and Stimulations

Mouse peritoneal macrophages were harvested as previously described (Deng et al., 2018). Mice aged 8–12 weeks were injected intraperitoneally with 3 ml sterile 3% thioglycollate broth, and macrophages were collected by peritoneal lavage 72 h later. Cells were resuspended in RPMI-1640 containing 10% fetal bovine serum (Gibco) and 1% antibiotics (Gibco) and plated in 12-well or 6-well plates overnight. Before stimulation, the cells were washed 3 times with sterile PBS and changed with FBS-free RPMI-1640. The cells were pretreated with 8-ol as indicated dose 1 h before being stimulated with LPS + HMGB1 complex. For some experiments, macrophages were primed with LPS (100 ng/ml) for 3 h and then pretreated with 8-ol (2.5 μM, 5 μM, and 10 μM) 1 h before the following indicated stimulations: ATP (5 mM) or nigericin (10 μM) was transfected into macrophages by Lipofectamine 3000 for 1 h, SiO_2_ (20 μg/ml) or MSU (400 μg/ml) was transfected into macrophages by Lipofectamine 3000 for 6 h, Flagellin (2 μg/ml) was transfected into macrophages by Lipofectamine 3000 for 1 h, and poly (dA:dT) (1 μg/ml) was transfected into macrophages by Lipofectamine 3000 for 16–18 h. Cell lysates and supernatants were collected for Western blots, ELISA, and LDH release. For the PI uptake assay, 5 μM PI was added at the indicated time after LPS+HMGB1 complex stimulation and measured using fluorescence at an excitation wavelength of 530 nm.

### RNAi Knockdown

For CASP4 siRNA knockdown, THP1 was primed with PMA (100 ng/ml) and seeded in 12-well plates. siRNA transfection was performed using the Lipofectamine RNAiMAX Transfection Reagent (Thermo Fisher Scientific, #13778150) by following the manufacturer’s instructions. The siRNA sequences are TCT​ACA​CTA​TAG​TCC​AGA​CCC (CASP4-1), GTC​TGG​ACT​ATA​GTG​TAG​ATG (CASP4-2), and CGT​ACG​CGG​AAT​ACT​TCG​A (control), which were described previously (Shi et al., 2014).72 h after transfection, the cells were stimulated with LPS alone or LPS+HMGB1 complex with or without indicated doses of 8-ol.

### Isolation of Cytosol Fraction and LPS Activity Assay

Subcellular fractionation of mouse peritoneal macrophages was conducted by using a digitonin-based fractionation method as described previously, with modifications (Vanaja et al., 2016). 4 × 10^6^ cells were stimulated with LPS (1 μg/ml) alone or LPS (1 μg/ml) + HMGB1 (400 ng/ml) in the presence or absence of 8-ol (2 μM) for 2 h. Then the cells were washed 3 times with sterile cold PBS and, subsequently, treated with 300 ul of 0.005% digitonin extraction buffer on ice for 10–15 min. After centrifugation at 13000 rpm at 4°C for 15 min, the supernatant was collected as the cytosol. The residual cell fraction was collected in 300 µl of 0.1% CHAPS buffer. Western blots for LAMP1, Na^+^/K^+^ ATPase, Rab7, and *β*-actin were performed to confirm the purity of the cytosol fraction.

### Proximity-Ligation Assay

The interaction between LPS and caspase-11 was analyzed using a proximity-ligation assay kit (Sigma 92008). Cells were cultured in a six-well glass dish in RPMI-1640, primed with LPS (100 ng/ml) for 4 h, and stimulated with LPS (5 μg/ml) alone or LPS (5 μg/ml) + HMGB1 (10 μg/ml) in the presence or absence of 8-ol (2 μM) for 2 h. Then the cells were washed with PBS 3 times, fixed with 4% formaldehyde for 15 min, and permeabilized with 0.1% Triton for 10 min. After washing and blocking with assay buffer for 1 h at room temperature, the primary antibody pair of different species directed to LPS (mouse monoclonal 2D7/1) and caspase-11 (rat monoclonal 17D9) was added and incubated at 4°C overnight. Then the proximity-ligation assay was carried out according to the manufacturer’s instructions. Images were taken using a Nikon Ni-U microscope and quantified using Image-J software.

### Mouse HMGB1 Homologous Modeling and Docking Calculation

The mouse HMGB1 model was prepared using the ModWeb server, which uses the MODELLER program in homology modeling. The box-A (PDB:4QR9) domain and the box-B (PDB:1HMF) domain from the crystal structure of mouse HMGB1 were used as the template structure to match the full length of HMGB1. Small-molecule (8-ol) docking was done using AutoDock with AutoDockTools. The model was defined as rigid, while the ligand was flexible. The docking energy between the small inhibitor 8-ol and HMGB1 is −7.99 kal/mol.

### Circular Dichroism (CD) Analysis

Circular dichroism spectra of different samples were obtained by using Jasco-815. Approximately 200 μL of the sample was detected by using a 1-mm quartz cell. The bandwidth was set to 2 nm, with a scanning speed of 200 nm min^−1^. All scans were taken from 190 to 260 nm, and all spectra were an average of 5 scans. Spectra measurement software was used to analyze the original data.

### Western Blots

Proteins in macrophage culture supernatants were precipitated with methanol/chloroform (4:1). Protein concentrations of cell and tissue lysates were measured by using the BCA method. All the samples were separated using 12% SDS-PAGE and subsequently transferred onto PVDF membranes (Millipore). All of the antibodies were used at 1:1000 dilution, except anti–caspase-11 Ab (1:500). Blots were normalized to *β*-actin expression.

### ELISA and LDH Assay

Cell culture supernatant samples were analyzed using an eBioscience Ready-SET-Go ELISA kit: IL-1α (#88-5019-77), IL-1β (#88-7013-77), TNF-α (#88-7324-77), IL-6 (#88-7064-77), human IL-1α ELISA MAX™ Deluxe (Biolegend, #445804), and human IL-1β ELISA MAX™ Deluxe (Biolegend, #437004). Cell death was measured by using an LDH Cytotoxicity Assay kit (Beyotime Biotechnology, C0017).

### Statistical Analysis

All data were analyzed using GraphPad Prism software (version 7.01) (GraphPad Software, Inc., La Jolla, CA, United States). Data were analyzed by using Student’s t-test, which was used for comparison between two groups, or one-way ANOVA followed by post hoc Bonferroni test, for multiple comparisons. Survival data were analyzed using the log-rank test. A *p*-value < 0.05 was considered statistically significant for all experiments. All values are presented as mean ± SD.

## Results

### A Phenotypic Screening Approach Identified 8-Hydroxyquinoline–Based Small Molecule as a Bioactive Inhibitor of the HMGB1–Caspase-11 Pathway

**FIGURE 1:**
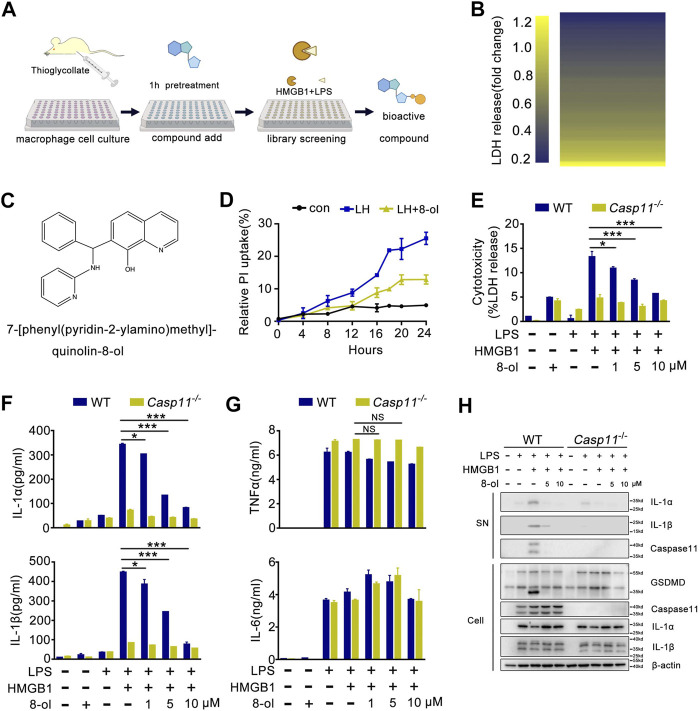
Phenotypic screening system identified that 8-ol specifically inhibits HMGB1-mediated caspase-11 signaling *in vitro*. **(A)** Schematics of the screening steps from compound library in mouse macrophage stimulated with recombinant HMGB1 plus LPS. **(B)** Heat map of LDH release changes in macrophages exposed to both recombinant HMGB1 (400 ng/ml) and LPS (1 μg/ml) for 16 h with 102 bioactive compounds (10 μM). **(C)** Chemical structure of 7-[phenyl (pyridin-2-ylamino) methyl] quinolin-8-ol. **(D)** PI uptake in a time-dependent manner in mouse macrophage stimulated with recombinant HMGB1 (400 ng/ml) and LPS (1 μg/ml) in the absence or presence of 8-ol (2 μM). **(E**–**G)** LDH and cytokine (IL-1α, IL-1β, TNFα, and IL-6) release from WT or *Casp11* ^*−/−*^ peritoneal macrophages stimulated with HMGB1 (400 ng/ml) plus LPS (1 μg/ml) for 16 h in the absence or presence of 8-ol of indicated dose. **(H)** Western blots for IL-1α, IL-1β, and caspase-11 in the supernatant or the cleavage of GSDMD and the expression of IL-1α, IL-1β, and caspase-11 in the cell lysates of WT or *Casp11* ^*−/−*^ peritoneal macrophages stimulated with HMGB1 (400 ng/ml) plus LPS(1 μg/ml) in the absence or presence of 8-ol for 16 h. Graphs show the mean ± SD of technical replicates and are representative of at least three independent experiments. *p < 0.05; **p < 0.01; ***p < 0.001, NS: not significant.

A prior study has demonstrated that recombinant HMGB1 mediated cytosolic translocation of LPS and induced caspase-11–dependent immune responses in sepsis and has uncovered that HMGB1 could be a potential pharmacological target for treating sepsis (Deng et al., 2018). In light of these findings, we established a novel screening system in which mouse peritoneal macrophages were incubated with recombinant HMGB1 plus LPS to identify bioactive compounds which could selectively inhibit the HMGB1–caspase-11 pathway (Figures 1A,B). By screening 102 compounds, we found 7-[phenyl (pyridin-2-ylamino)methyl]quinolin-8-ol (8-ol) (Figure 1C), a novel 8-hydroxyquinoline–based small molecule that significantly inhibits caspase-11–dependent pyroptotic cell death (Figures 1D,E) and caspase-11–dependent release of IL-1α and IL-1β in a concentration-dependent manner (Figure 1F). However, addition of 8-ol does not affect the release of TNF and IL-6 (Figure 1G), suggesting that 8-ol does not attenuate the global inflammatory response. Western blot analysis revealed that 8-ol obviously attenuates the activation of IL-1α, IL-1β, and caspase-11 in the supernatant of WT mouse peritoneal macrophages and significantly abolishes the cleavage of GSDMD, the specific substrate of caspase-11 (Shi et al., 2015) in the cell lysates, but fails to inhibit the expression of caspase-11, IL-1α, IL-1β, and GSDMD in the cell lysates (Figure 1H). TLR4 signaling is required for the expression of caspase-11, and genetic deletion of TLR4 almost blocked the expression of caspase-11; however, 8-ol treatment did not impact the expression of caspase-11 in LPS-treated macrophages and endotoxemia mice (Supplementary Figure S1), which indicated that 8-ol has no effect on TLR4 signaling and specifically inhibits the HMGB1-mediated caspase-11–dependent pathway.

### 8-ol Does Not Inhibit Canonical Inflammasome Activation

**FIGURE 2:**
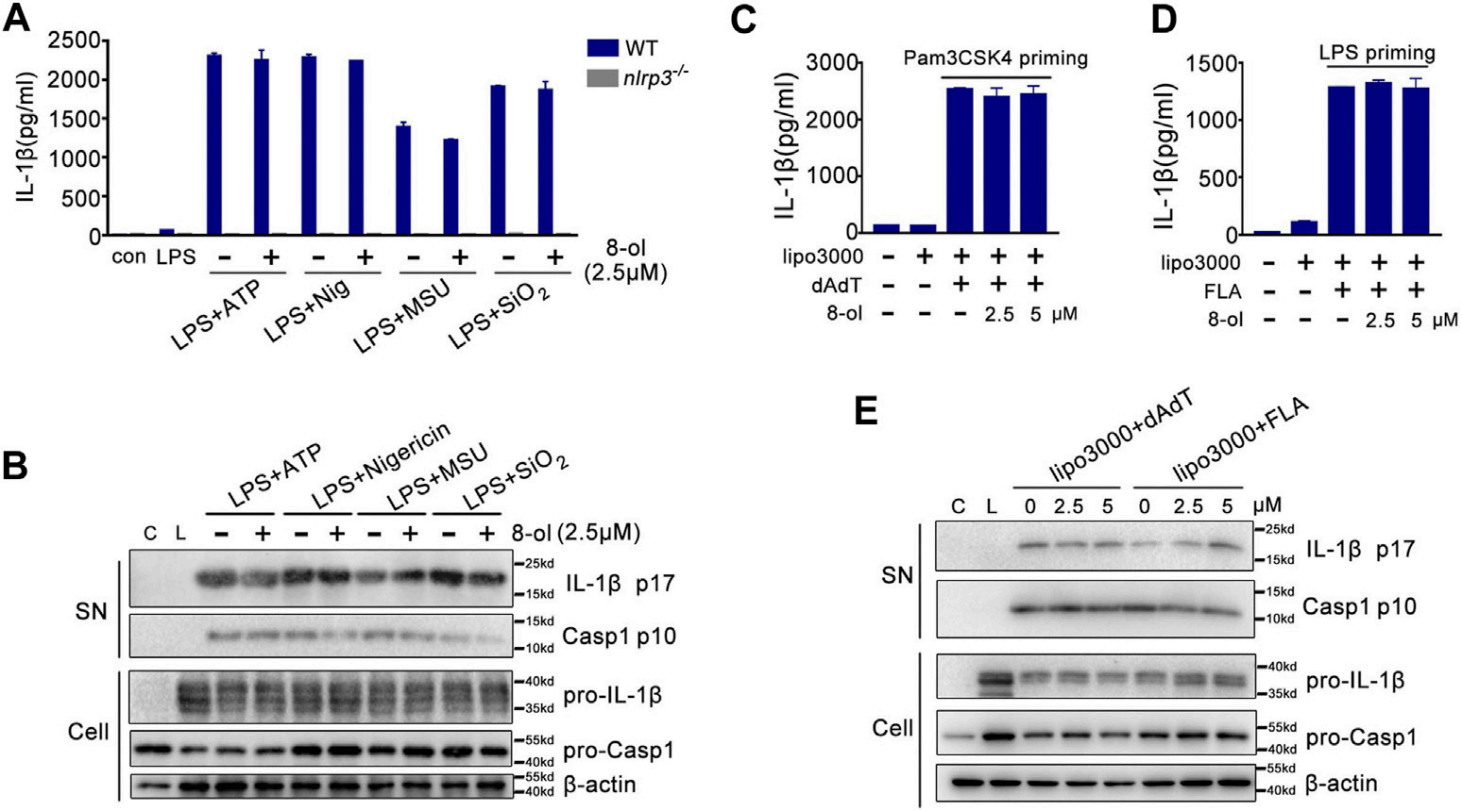
8-ol does not affect the activation of NLRP3, AIM2, and NLRC4 inflammasomes. **(A–B)** ELISA for IL-1β and Western blots for the cleavage of IL-1β and caspase-1 or the expression of IL-1β and caspase-1 from WT or *nlrp3* ^*−/−*^ LPS-primed (100 ng/ml) peritoneal macrophages stimulated with ATP, nigericin (Nig), monosodium urate crystals (MSU), and silicon crystals (SiO_2_) in the presence or absence of 8-ol (2.5 μM). **(C–E)** ELISA for IL-1β and Western blots for the cleavage of IL-1β and caspase-1 in the supernatant or the expression of IL-1β and caspase-1 in the cell lysates from Pam3CSK4 (1 μg/ml) or LPS (0.1 μg/ml)-primed peritoneal macrophages which are then transfected with 1 μg/ml poly (dA:dT) or 2 μg/ml flagellin by Lipofectamine 3000 in the presence or absence of 8-ol. Graphs show the mean ± SD of technical replicates and are representative of at least three independent experiments.

Caspase-11 is a receptor of cytosolic LPS and triggers noncanonical inflammasome activation (Kayagaki et al., 2011; Kayagaki et al., 2013; Shi et al., 2014; Kayagaki et al., 2015). Since 8-ol could specifically inhibit caspase-11–dependent pyroptotic cell death, next, we examined whether 8-ol influences other inflammasomes’ activation including NLRP3, AIM2, and NLRC4 signaling. Mouse peritoneal macrophages were primed with LPS, and NLRP3 stimulations including ATP, nigericin, silicon crystals (SiO_2_), and monosodium urate (MSU) crystals were added. 8-ol does not inhibit the release of IL-1β or the cleavage of caspase-1 and IL-1β in the supernatant from mouse primary macrophages even at the highest dose of 10 μM (Figures 2A,B; Supplementary Figure S2). Similar observations were made in the AIM2-dependent inflammasome activation induced by poly (dA:dT) double-stranded DNA and the NLRC4-dependent inflammasome activation engaged by flagellin (Figures 2C,D,E; Supplementary Figure S2).Taken together, 8-ol is a specific inhibitor of the HMGB1–caspase-11–GSDMD pathway.

### 8-ol Inhibits HMGB1–Caspase-4–Dependent Pyroptosis in Human Monocytic THP-1 Cells

**FIGURE 3:**
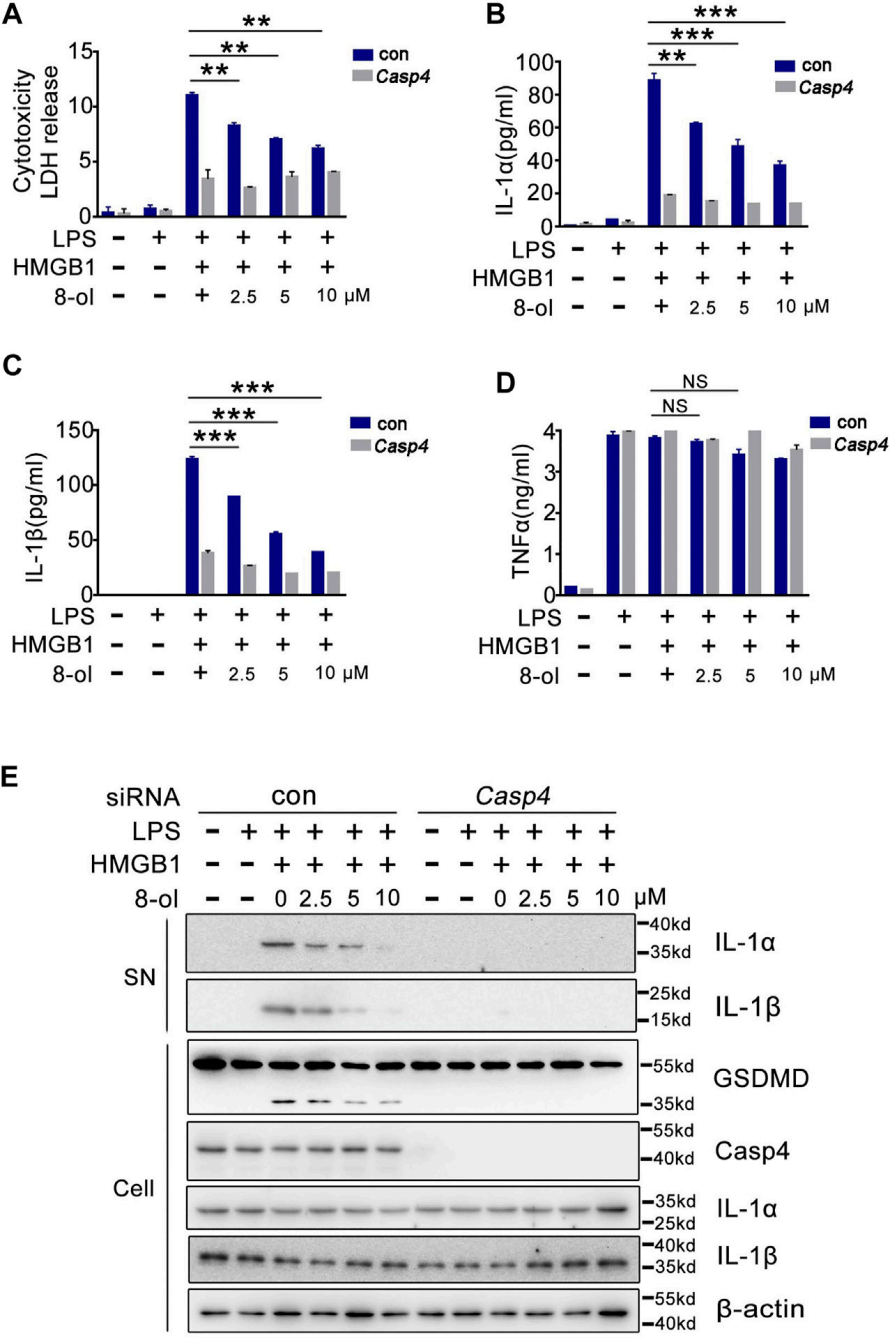
8-ol inhibits HMGB1-mediated caspase-4–dependent pyroptosis in human monocytes. **(A–D)** LDH and cytokine (IL-1α, IL-1β, and TNFα) release from PMA-primed human monocytic THP-1 cells transfected with scrambled siRNA or CASP4-specific siRNA upon HMGB1 (400 ng/ml) and LPS (1 μg/ml) stimulation in the presence or absence of 8-ol. **(E)** Western blots for IL-1α and IL-1β in the supernatant or the cleavage of GSDMD and the expression of caspase-4, IL-1α, and IL-1β in the cell lysates stimulated with or without the indicated dose of 8-ol. Graphs show the mean ± SD of technical replicates and are representative of at least three independent experiments. *p < 0.05; **p < 0.01; ***p < 0.001, NS: not significant.

Humans do not possess caspase-11. One of the enzymes most homologous to caspase-11 is caspase-4 (Kayagaki et al., 2011). Next, we examined whether 8-ol could inhibit HMGB1–caspase-4–dependent immune response in human cells. Consistent with our previous findings (Shi et al., 2014), small interference RNA (siRNA) knockdown of caspase-4 strongly inhibits HMGB1-induced LDH, IL-1α, and IL-1β release in combination with LPS from THP1 cells, and 8-ol does not affect the release of TNF (Figure 3A–D). Western blots show that 8-ol obviously suppresses the activation of IL-1α and IL-1β in the supernatant and the cleavage of GSDMD in the cell lysates, but it did not affect the expression of IL-1α, IL-1β, caspase-4, and GSDMD (Figure 3E).

### 8-ol Inhibits HMGB1-Mediated Cytosolic Delivery of LPS

**FIGURE 4:**
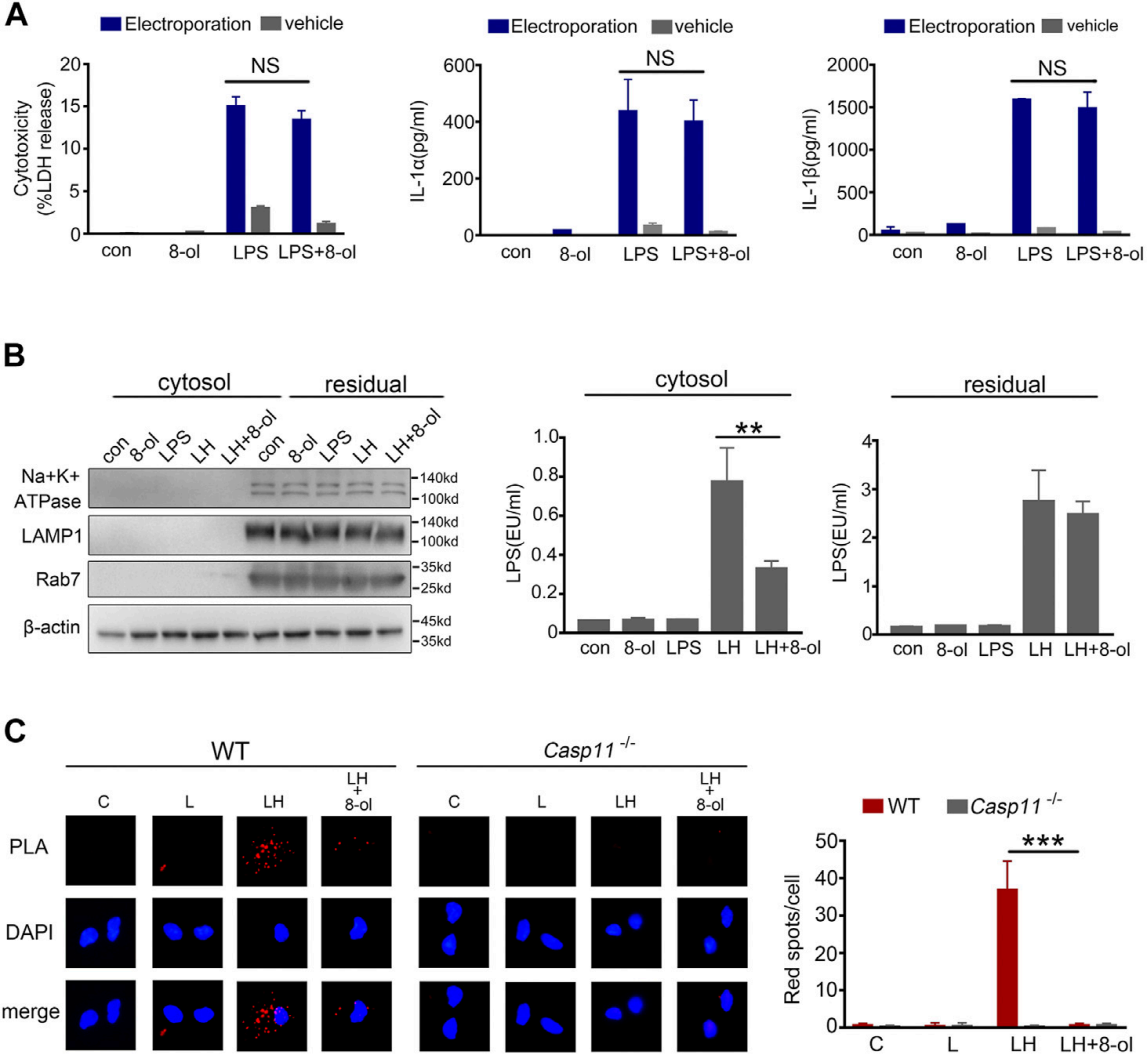
8-ol prevents HMGB1-mediated cytoplasmic translocation of LPS. **(A)** LDH, IL-1α, and IL-1β release from mouse peritoneal macrophages upon LPS stimulation with or without 8-ol electroporation. **(B)** Western blots for Na^+^/K^+^ ATPase, LAMP1, Rab7, and *β*-actin, LAL assay for LPS (EU, endotoxin units) in the cytoplasmic and residual fractions of mouse peritoneal macrophages treated with LPS alone (1 μg/ml) or LPS (1 μg/ml)+HMGB1 (400 ng/ml) with or without 8-ol for 2 h. **(C)** Physical interaction between LPS and caspase-11 was visualized as red spots using the proximity-ligation assay (PLA). Mouse peritoneal macrophages were primed with LPS (100 ng/ml) for 4 h and then stimulated with LPS (1 μg/ml) or LPS (1 μg/ml) + HMGB1 (400 ng/ml) with or without 8-ol for 2 h. Graphs show the mean ± SD of technical replicates and are representative of at least three independent experiments. *p < 0.05; **p < 0.01; ***p < 0.001, NS: not significant.

Cytoplasmic LPS triggers caspase-11 activation and induces pyroptosis (Shi et al., 2014). Our previous study showed that HMGB1 could deliver LPS into the cytosol for caspase-11 activation (Shi et al., 2014). Next, we investigated whether 8-ol can inhibit the process. The LPS level is measured by a digitonin-based cytosolic fraction assay. The results show that addition of 8-ol significantly reduces the cytosolic translocation of LPS in mouse macrophages treated with recombinant HMGB1 plus LPS (Figure 4B).To confirm the phenomenon, the proximity-ligation assay (PLA) was used to measure the combination of LPS and caspase-11 stimulated with LPS alone or LPS plus HMGB1 with or without 8-ol. The results show markedly fewer bindings of LPS with caspase-11 when cells were treated with 8-ol (Figure 4C). However, when we artificially delivered LPS directly to the cytosol of mouse macrophages by electroporation, the addition of 8-ol failed to inhibit caspase-11–dependent release of LDH, IL-1α, and IL-1β (Figure 4A). Taken together, 8-ol prevents caspase-11 activation by inhibiting HMGB1-mediated cytosolic delivery of LPS.

### 8-ol Suppresses HMGB1–LPS Binding by Directly Targeting HMGB1 to Change the Secondary Conformation

**FIGURE 5:**
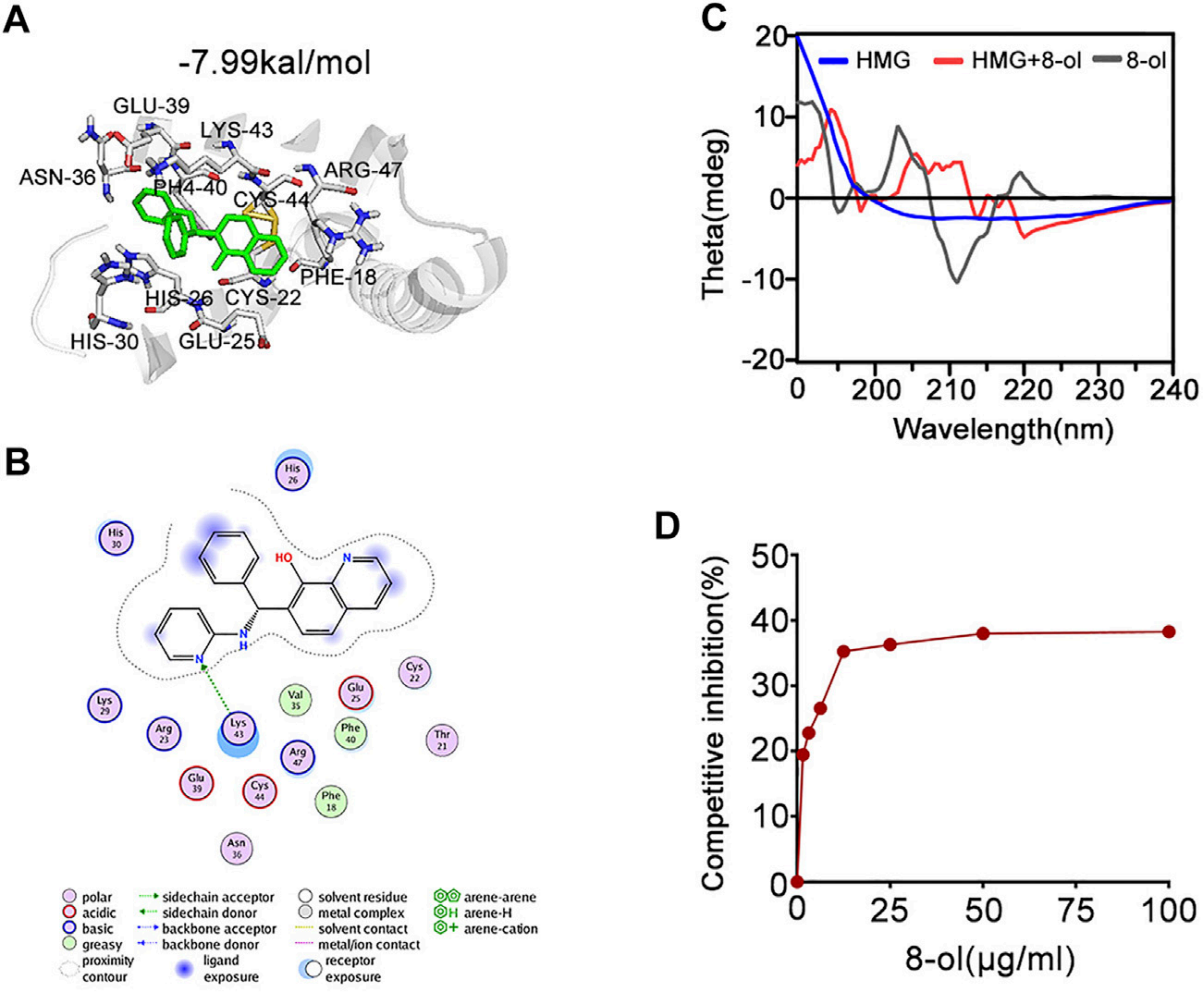
8-ol targets HMGB1 and changes the secondary conformation and disturbs the binding with LPS. **(A)** 3D binding mode diagrams between HMGB1 and the chemical 7-PMQ-8-ol. The protein is shown in the cartoon and colored in gray, and the chemical is colored in green. The key amino acid residues were shown as sticks. Docking score is −7.99 kcal/mol. **(B)** 2D binding mode illustrates the key amino acid residues in the ligand–protein complexes. **(C)** CD spectra of the chemical 8-ol between HMGB1. **(D)** The LPS-binding capacity of HMGB1 incubated with different concentrations of the chemical 8-ol.

Next, we examined the mechanisms by which 8-ol inhibits HMGB1-mediated cytosolic translocation of LPS. Molecular interactions of 8-ol and its receptor HMGB1 were calculated by using AutoDock4. Analysis shows that 8-ol binds to HMGB1 with −7.99 kal/mol binding energy (Figures 5A,B). The interaction residues of HMGB1 with 8-ol, including PHE18, CYS44, ARG47, LYS43, PHE40, GLU39, ASN36, CYS22, GLU25, HIS26, and HIS30 (Figures 5A,B), suggest that there is a high affinity binding of 8-ol with HMGB1. HMGB1–LPS binding is essential for the cytosolic delivery of LPS. We hypothesized that 8-ol could disrupt the interaction between LPS and HMGB1. We employed circular dichroism (CD) spectra to investigate whether 8-ol has a direct impact on HMGB1. The results showed that the emission wavelength of HMGB1 was significantly changed, and the 8-ol–induced changing of the typical alpha-helix leads to the momentous structural alterations of the HMGB1 protein (Figure 5C). However, 8-ol does not change the secondary conformation of another well-known LPS binding protein, LPB (Supplementary Figure S4), indicating that 8-ol could specifically interact with HMGB1 and disrupt the interaction between LPS and HMGB1. Furthermore, we studied a competitive enzyme-linked immunosorbent assay to confirm that 8-ol could inhibit HMGB1–LPS interaction in a dose-dependent manner (Figure 5D). Taken together, 8-ol could directly target HMGB1 and change the secondary structure of HMGB1 to inhibit the interaction between LPS and HMGB1.

### 8-ol Protects Mice Against Caspase-11–Mediated Lethality in Endotoxemia and Bacterial Sepsis

**FIGURE 6:**
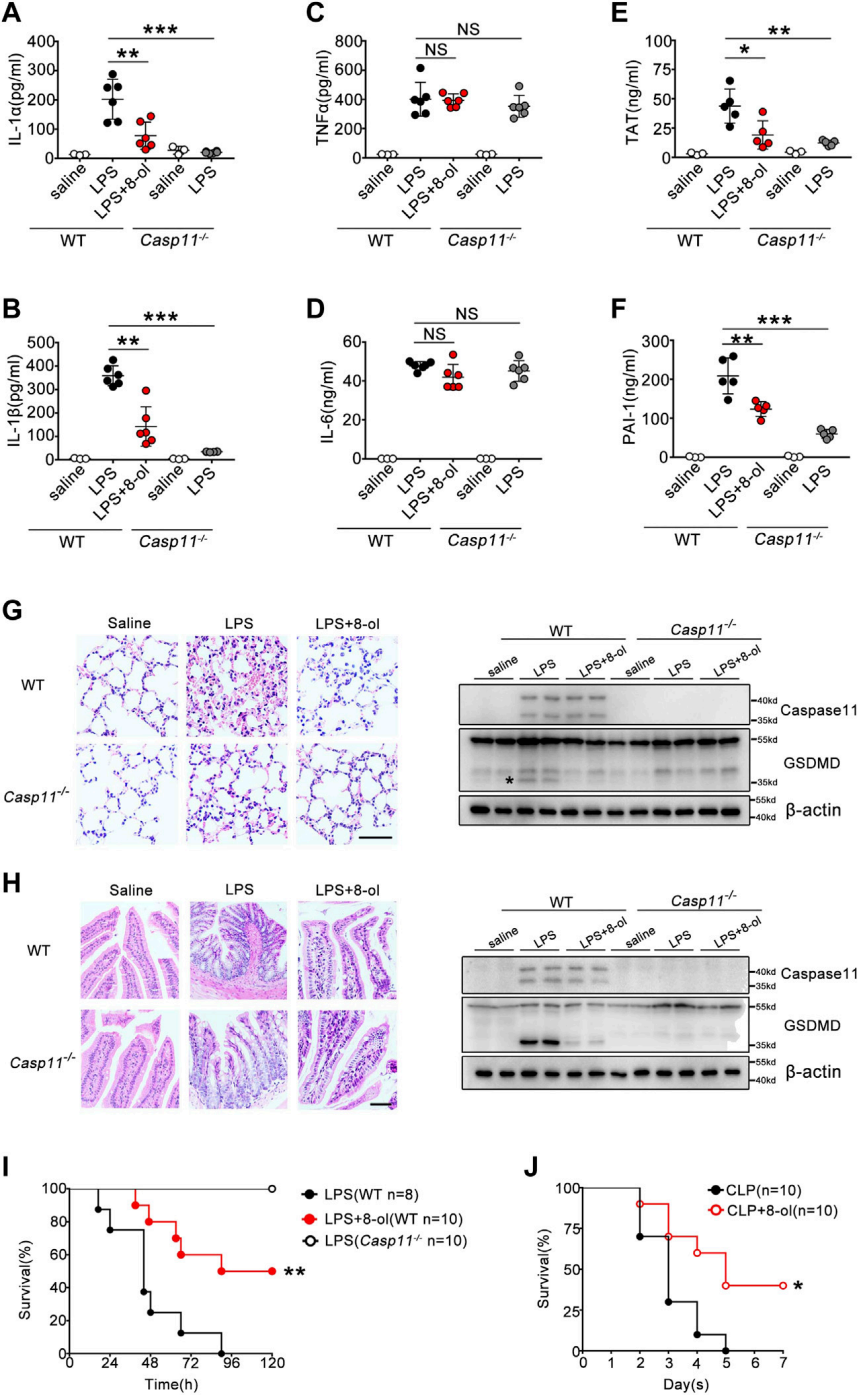
8-ol inhibits caspase-11–mediated immune responses *in vivo*. **(A**–**D)** IL-1α, IL-1β, TNFα, and IL-6 release in the serum was measured by ELISA. WT or *Casp11* ^*−/−*^ mice were pretreated with 8-ol (4 mg/kg) by intraperitoneal injection 30 min before being intraperitoneally challenged with LPS (20 mg/kg). **(E,F)** Plasma concentrations of TAT complexes and PAI-1 were measured at 16 h in mice injected with LPS (20 mg/kg) with or without 4 mg/kg 8-ol administration. **(G,H)** H&E staining shows representative images of lung and intestine tissues of endotoxemic WT or *Casp11* ^*−/−*^ mice (left panel). Scar bar: 50 μm. Western blots for the cleavage of GSDMD and the expression of caspase-11 in the lung and intestine. WT or *Casp11* ^*−/−*^ mice were pretreated with 8-ol (4 mg/kg) by intraperitoneal injection 30 min before being intraperitoneally challenged with LPS (20 mg/kg). “*”: GSDMD cleavage. **(I,J)** WT or *Casp11* ^*−/−*^ mice were pretreated with 8-ol (4 mg/kg) 30 min before being intraperitoneally challenged with LPS (20 mg/kg) or subjected to cecum ligation and puncture (CLP) 1 h after 8-ol (4 mg/kg) treatment. Survival rates were shown by Kaplan–Meier survival curves. Circles represent individual mice. *p < 0.05; **p < 0.01; ***p < 0.001; NS: not significant (Student’s t test and log-rank test for survival).

Caspase-11 is crucial for organisms to defend themselves against LPS-induced lethality in endotoxemia or bacterial sepsis. Next, we investigated whether 8-ol exhibits a caspase-11–dependent protectiveness *in vivo*. WT and caspase-11–deficient mice were subjected to lethal injection of LPS with or without 8-ol treatment. Caspase-11–dependent release of IL-1α and IL-1β was significantly reduced by 8-ol treatment. Administration of 8-ol does not alter the release of TNF and IL-6 (Figures 6A–D), in accordance with our *in vitro* experiments. To further investigate whether 8-ol could inhibit caspase-11 signaling *in vivo*, we detected the cleavage of GSDMD, the enzyme substrates of activated caspase-11 in lung and intestine tissues. Treatment with 8-ol significantly inhibits the cleavage of GSDMD in endotoxemia mice (Figures 6G,H). Furthermore, administration of 8-ol markedly attenuated caspase-11–dependent lung and intestine injury (Figures 6G,H) and significantly promoted survival in the lethal endotoxemia and CLP sepsis model (Figures 6I,J). Recent research in our group showed a novel link between caspase-11 signaling and the consequent coagulation and lethality during endotoxemia and bacterial sepsis ((Kayagaki et al., 2011; Kayagaki et al., 2013; Shi et al., 2014; Kayagaki et al., 2015). Thrombin–antithrombin (TAT) complexes and plasminogen activator inhibitor type-1 (PAI-1) increased in circulation during endotoxemia. In line with this finding, TAT complex and PAI-1 increased remarkably in WT mice compared with *Casp11* ^*−/−*^ mice challenged by a high dose of LPS. Treatment with 4 mg/kg 8-ol markedly reduced the secretion of TAT and PAI-1 in the plasma of WT mice (Figures 6E,F), which indicated that 8-ol could prevent caspase-11–dependent DIC in endotoxemia. To further confirm that 8-ol inhibits caspase-11 activation *in vivo*, we used the cecum ligation and puncture (CLP) model, a clinically relevant murine model of Gram-negative polymicrobial sepsis, and observed that 8-ol treatment markedly inhibited caspase-11–dependent release of IL-1α and IL-1β. Furthermore, administration of 8-ol prevented caspase-11–mediated GSDMD cleavage in the lung tissues and markedly attenuated caspase-11–dependent lung injury after CLP (Supplementary Figure S3). In summary, these data suggest that 8-ol might be of use in the treatment of LPS-induced endotoxemia and CLP-induced Gram-negative polymicrobial sepsis.

## Discussion

Sepsis, caused by severe infection, is commonly accompanied by organ dysfunction or death. Currently, anti-infection and life-supporting techniques have been the most common strategy for treating sepsis in recent decades. However, sepsis is still a leading cause of in-hospital mortality (Cecconi et al., 2018). Although understanding of the pathophysiology of sepsis is largely developed in the new century (Rice and Bernard, 2005), a specific medicine is not available in clinics. Thus, the development of targeted therapies is urgent in the treatment of sepsis. In this study, we establish a novel screening strategy using recombinant HMGB1 and LPS from mouse peritoneal macrophages and identify that 8-ol specifically inhibits HMGB1-mediated caspase-11 signaling. Treatment with 8-ol robustly prevents caspase-11–dependent DIC and lethality in endotoxemia or sepsis.

The majority of sepsis cases are attributed to Gram-negative bacteria, in which lipopolysaccharide (LPS) is the main pathological component of the membrane. Clinical studies have revealed that increased concentration of LPS in severe sepsis is associated with higher mortality, and removal of LPS from the circulation improves the outcome of Gram-negative sepsis (Brandtzaeg et al., 1989; Opal et al., 1999; Marshall et al., 2004; Davies and Cohen, 2011). As the intracellular LPS receptor, caspase-11 is a key component of the innate immune response to Gram-negative bacterial infection. Previous studies have shown that caspase-11 signaling activation mediates disseminated intravascular coagulation (DIC), broad organ injury and dysfunction, and lethality in bacterial sepsis. Collectively, inhibiting caspase-11 signaling should be a promising strategy in the treatment of sepsis. Our previous study bridged HMGB1 and LPS internalization, which answers the question of how LPS transfers into the cytosol in caspase-11 activation. Thus, using HMGB1 plus LPS for screening the effective and specific inhibitor of caspase-11 signaling would be promising in the development of medicine for treating sepsis.

High mobility group box 1 (HMGB1) is a highly conserved nuclear protein which is widely distributed in mammalian cells. Its multiple functions include DNA stabilization and repairing, coagulation and fibrinolysis system regulation, and more. With the discovery of its late pro-inflammatory effect, HMGB1 has become one of the hot spots of critical care medicine research in recent years (Goodwin and Johns, 1973). However, it would be harmful when released into the cytosol or extracellular circulation in pathological conditions, such as endotoxemia or sepsis (Wang et al., 1999; Wang et al., 2004; Lamkanfi et al., 2010; Lu et al., 2012). Previously, we found that type I interferon mediates hepatocytes to release HMGB1 which facilitates LPS internalization for caspase-11 activation and consequent coagulation cascades (Yang et al., 2020). It has been evidenced that circulating levels of HMGB1 are boosted in patients with sepsis (Antoine et al., 2020), and septic patients with a higher level of HMGB1 are more susceptible to DIC and organ dysfunction. Moreover, genetic deletion of HMGB1 or neutralizing circulating HMGB1 confers protection in lethal endotoxemia and bacterial sepsis (Wang et al., 1999; Wang et al., 2004; Lamkanfi et al., 2010; Andersson and Tracey, 2011).

In the present study, 8-ol significantly inhibits HMGB1/LPS–mediated caspase-11 signaling by suppressing the cytosolic transfer of LPS. Given the critical role of HMGB1/LPS binding in LPS internalization, we employed circular dichroism (CD) spectra to investigate whether 8-ol inhibits the binding of HMGB1 and LPS by affecting the construction of HMGB1. The presence of 8-ol significantly changes the secondary conformation of HMGB1 and competitively inhibits the binding of LPS and HMGB1. Thus, targeting the HMGB1 conformation that attenuates the binding with LPS would be a potential approach in the treatment of sepsis.

As a derivative of 8-HQ that is an attractive scaffold for designing BoNT/A (botulinum neurotoxin A light chain) inhibitors (Caglič et al., 2014), 8-ol plays a novel and surprising role in the treatment of sepsis. Some kind of 8-HQ derivatives have been clinically approved for the medicinal purposes, such as clioquinol, an antiseptic for an oral intestinal amebicide. (Caglič et al., 2014; Bareggi and Cornelli, 2012). Because of the good *in vivo* properties and long drug half-life of clioquinol, it has been successfully used to improve the diseased state of Alzheimer’s patients in a pilot phase 2 clinical trial (Ritchie et al., 2003). Consequently, the safety and drug ability of 8-ol would be proposed, although further validation should be conducted. In addition, it remains to be investigated whether other derivatives of 8-HQ have similar effects on the inhibition of HMGB1/LPS-mediated caspase-11 signaling and the corresponding organ dysfunction and death in sepsis. Thus, our study constitutes a profound screening system that develops an effective chemical for preventing sepsis, which may provide a novel target and new medicine in the treatment of sepsis.

## Data Availability

The original contributions presented in the study are included in the article/Supplementary Material; further inquiries can be directed to the corresponding author.
