# Supplementary material for: An 8-Hydroxy-Quinoline Derivative Protects Against Lipopolysaccharide-Induced Lethality in Endotoxemia by Inhibiting HMGB1-Mediated Caspase-11 Signaling
Source: Front Pharmacol. 2021 May 21;12:673818. doi: 10.3389/fphar.2021.673818 (PMC8176522; doi:10.3389/fphar.2021.673818)
Supplement: Supplementary file 1 [file DataSheet1.pdf]

Supplementary Figures

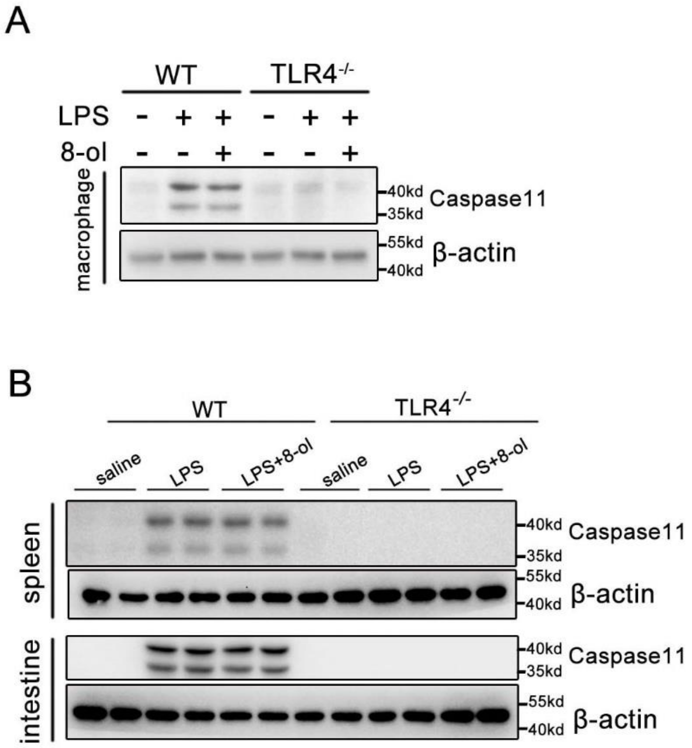

Figure S1 The effect of 8-ol on TLR4 signaling

Immuno-blot to detect the casepase11 (Casp11) expression (A)in the cell lysates treated with LPS 0.1µg/ml alone or LPS 0.1µg/mL + 8-ol 2.5µM from WT or *TLR4*$^{-/-}$ mice peritoneal macrophages or (B) in intestine or spleen from WT or *TLR4*$^{-/-}$ mice injected intraperitoneally with LPS (20mg/kg) or LPS (20mg/kg) + 8-ol(4mg/kg).

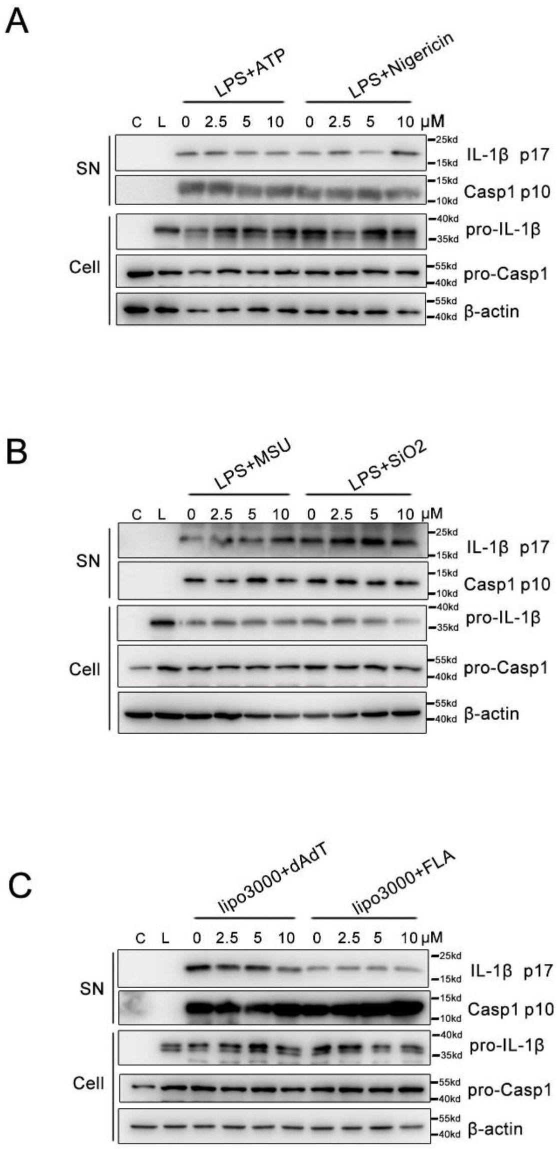

Figure S2 8-ol does not inhibit the activation of canonical inflammasomes

(A-B) Western blots for the cleavage of IL-1β and caspase-1 in the supernatant or the expression of IL-1β , caspase-1 in the cell lysates from LPS primed (100ng/mL) peritoneal macrophages stimulated with ATP, nigericin(Nig), monosodium urate crystals(MSU) and silicon crystals(SiO_2) in the presence or not of different doses of 8-ol(2.5μM,5μM,10μM).

(C) Western blots for the cleavage of IL-1β and caspase-1 in the supernatant or the expression of IL-1β, caspase-1 in the cell lysates from Pam3CSK4(1μg/mL) or LPS(0.1μg/mL) primed peritoneal macrophages then transfected with 1μg/mL poly(dA:dT) or 2μg/mL flagellin in the presence or not of different doses of 8-ol(2.5μM,5μM,10μM).

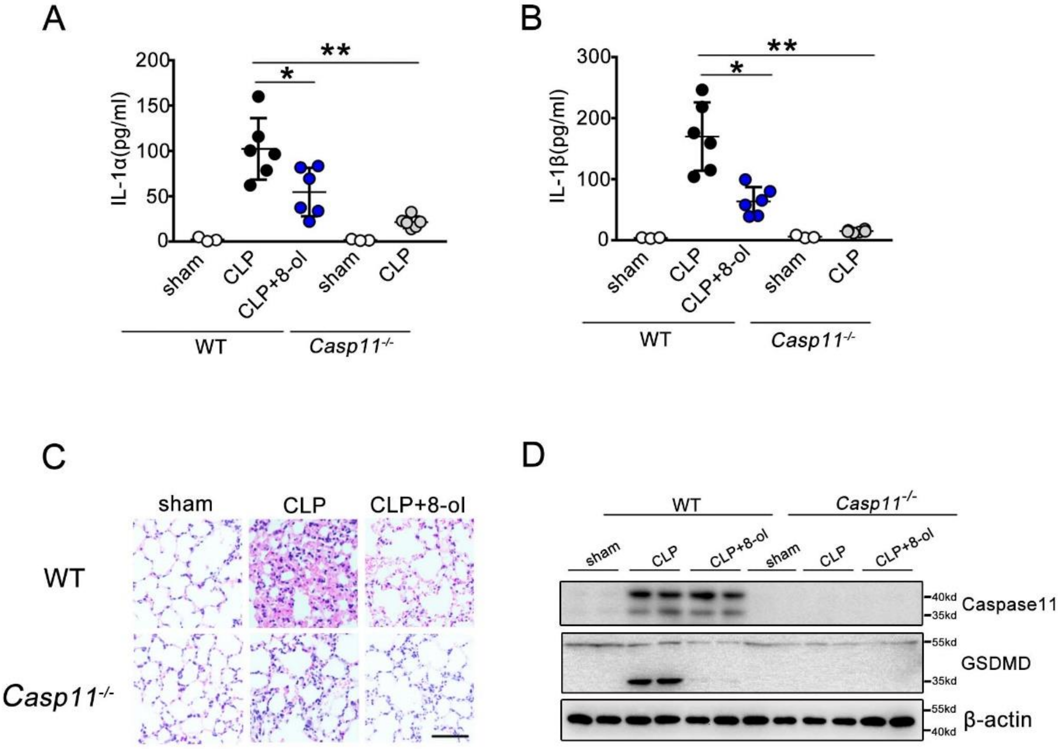

Figure S3 8-ol attenuated caspase-11-dependent immune responses in CLP sepsis mice

(A-D) WT or *Casp11*$^{-/-}$ mice were treated with 8-ol 4mg/kg by intraperitoneal injection 1h after subjected to cecum ligation and puncture (CLP) or sham operation. Serum levels of IL-1α,IL-1β *(A)* , hematoxylin and eosin (H&E) stained sections of lung*(C)*, Scar bar:50μm. Western blots for GSDMD-NT, Caspase11 in lung*(D)*.

Circles represent individual mice. $*p < 0.05$; $**p < 0.01$.

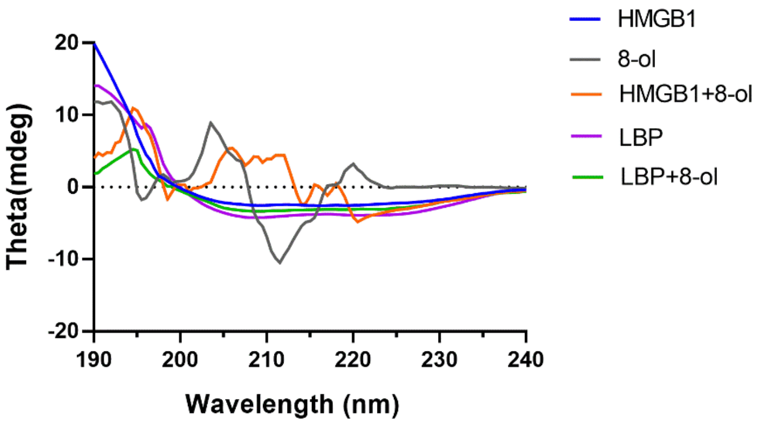

Figure S4 CD spectra of the chemical 8-ol between HMGB1 and LBP
